# Recombinant biosynthesis of bacterial cellulose in genetically modified *Escherichia coli*

**DOI:** 10.1007/s00449-017-1864-1

**Published:** 2017-11-24

**Authors:** Gizem Buldum, Alexander Bismarck, Athanasios Mantalaris

**Affiliations:** 1 Biological Systems Engineering Laboratory (BSEL), Department of Chemical Engineering, Imperial College London, South Kensington Campus, London, SW7 2AZ UK; 2 Polymer and Composite Engineering (PaCE) Group, Department of Chemical Engineering, Imperial College London, South Kensington Campus, London, SW7 2AZ UK; 3 Polymer and Composite Engineering (PaCE) Group, Institute of Materials Chemistry and Research, Faculty of Chemistry, University of Vienna, Währinger Str. 42, 1090 Vienna, Austria; 4 Department of Bioengineering, Marmara University, Göztepe Campus, Istanbul, Turkey

**Keywords:** Bacterial cellulose, *Escherichia coli*, Recombinant biosynthesis, Optimization of culture conditions, Plasmid maintenance

## Abstract

Bacterial cellulose (BC) exhibits unique properties such as high purity compared to plant-based cellulose; however, commercial production of BC has remained a challenge, primarily due to the strain properties of cellulose-producing bacteria. Herein, we developed a functional and stable BC production system in genetically modified (GM) *Escherichia coli* by recombinant expression of both the BC synthase operon (*bcs*ABCD) and the upstream operon (*cmcax, ccp* *Ax*). BC production was achieved in GM HMS174 (DE3) and in GM C41 (DE3) by optimization of the culture temperature (22 °C, 30 °C, and 37 °C) and IPTG concentration. BC biosynthesis was detected much earlier in GM C41 (DE3) cultures (3 h after IPTG induction) than those of *Gluconacetobacter hansenii*. GM HMS174 (DE3) produced dense fibres having a length of approximately 1000–3000 μm and a diameter of 10–20 μm, which were remarkably larger than the fibres of BC typically produced by *G. hansenii*.

**Electronic supplementary material:**

The online version of this article (10.1007/s00449-017-1864-1) contains supplementary material, which is available to authorized users.

## Introduction

Bacterial cellulose (BC) is a biomaterial with unique properties and receiving a growing attention for industrial applications. BC has many advantages over plant-based cellulose such as high purity, which allows a purification process that is straightforward [1, 2]. In addition, it exhibits excellent mechanical and chemical properties including a high crystallinity, high tensile strength, high water holding capacity, high modulus of elasticity, and biocompatibility [3–5]. These properties enable BC to be used in a wide range of industrial applications such as medical products, electronics, paper, food, and cosmetics. BC provides all requirements for wound dressing material such as high porosity and providing a barrier against infection [6, 7].

Currently, plant-derived cellulose has a significant economic value with $200 billion per year worldwide. In 2016, the price of wood pulp, the main source of plant-based cellulose, was as low as $0.96 per kg. However, the extraction process of cellulose from wood causes both environmental hazards and requires high energy [8]. Recent developments have demonstrated the use of cellulose-producing bacteria to alleviate the demand on plant cellulose; nonetheless, the average cost of bacterial cellulose remains at $35 per pound ($77 per kg), which is still 80 times higher than plant-derived cellulose [9].

*Gluconacetobacter hansenii* (formerly known as *Gluconacetobacter xylinus*) is the most efficient BC producer among cellulose producer species [10]. Biosynthesis of cellulose is encoded by the bacterial cellulose synthesis (*bcs*) operon in *G. hansenii*. This operon is essential for a complete BC biosynthesis and consists of four subunits (*bcs*A, *bcs*B, *bcs*C, and *bcs*D). The first gene of the *bcs*ABCD operon, *bcs*A, encodes the catalytic subunit of cellulose synthase. The second gene, *bcs*B, encodes the regulatory subunit of cellulose synthase that binds to c-di-GMP, which plays an important role as the second messenger to activate the cellulose synthesis process [11]. Although the functions of *bcs*C and *bcs*D have not been completely clarified yet, it has been suggested that *bcs*C is responsible for the formation of pores in the membrane to secrete cellulose, as it encodes a protein similar to the proteins involved in the formation of membrane channels or pores [12]. Inactivation of *bcs*A, *bcsB*, or *bcs*C completely inhibits BC synthesis, indicating that these genes are crucial for the biosynthesis. In contrast, the inactivation of *bcs*D reduces BC production by 40%, which suggests that *bcs*D is involved in the crystallization of cellulose into nanofibrils [12–14].

Immediately upstream of the *bcs* operon, two more genes are encoded: *cmcax* and *ccpAx*. The endo-β-1,4-glucanase enzyme, which has cellulose hydrolyzing activity and enhances cellulose synthesis, is encoded by *cmcax* [15–17]. Based on an electron microscopic analysis, it has been suggested that endo-β-1,4-glucanase could have an influence on the ribbon assembly. CcpAx is also essential for BC production and plays a critical role in locating the *bcs* complex on the cell membrane [15]. It has been suggested that the functions of CcpAx could be related to extracellular transport of cellulose chains from the sites of polymerization within the cell membrane and the crystallization of cellulose fibrils [18, 19].

The progress made in BC production has been extensively discussed and the challenges of commercial scale BC production have been reviewed in our previous study [20]. Several strategies including the designing of advanced reactors and utilization of various carbon sources have been developed for enhanced production of BC [21]. So far, the most extensively studied bacteria are *G. hansenii*. However, the challenges in BC production are mainly related to the strain properties of *G. hansenii*: (1) slow growth compared to many other bacteria (such as *E. coli*), (2) low productivity, (3) susceptibility to culture conditions, and (4) the spontaneous mutation of cellulose-producing bacteria into cellulose non-producing mutants. Although BC biosynthesis is a highly complex process, novel cellulose-producing strains needs to be developed for enhanced BC production [21, 22].

*Escherichia coli*, as a well-characterized cell factory, represents a strong candidate for accomplishing BC production in new platforms due to its rapid growth kinetics. *E. coli* B and *E. coli* K12 lines are used almost equally as hosts for recombinant protein production. *E. coli* BL21, a B line derivative, is one of the most commonly used strains in research. This is due to the fact that BL21 strains are deficient in the production of proteases Lon and OmpT, leading to reduced recombinant protein degradation and higher yields [23–25]. HMS174 (DE3), an engineered successor of the *E. coli* K-12 wild-type, provides a recA1 mutation in a K-12 background. This mutation has a positive effect on plasmid stability [26]. After lysogenization with the DE3 prophage, it became a popular strain for the overexpression of heterologous proteins [27]. However, overexpression of membrane-associated proteins is often toxic to the cells and this can inhibit biomass formation and severely reduce product yields. C41 (DE3), derived from BL21 (DE3), allows high expression of a wide variety of toxic proteins that have previously been difficult or even impossible to express in bacteria [28–30]. The mutations of this strain that were reported to be related to *lac*UV5 operon or expression of T7 RNA polymerase prevented cell death associated with expression of many toxic recombinant proteins [31].

In this study, simultaneous expression of the bacterial cellulose synthase operon (*bcs*ABCD) and its upstream operon (*cmc*ax and *ccp* *Ax*) was performed by co-expression plasmids, pBCS and pCMP, respectively. The design of the vector constructs compatible with different *E. coli* strains has allowed us the BC production to be examined in these strains previously mentioned: BL21 (DE3), HMS174 (DE3), and C41 (DE3). Plasmid DNA maintenance and replication commonly create a metabolic burden in *E. coli* and the response of cells under this energy limiting condition is extremely complex, leading to the alterations of physiology and metabolism of host cells [32]. In return, this results in lower yields of plasmid DNA, particularly a decrease in process outcome. The maintenance of recombinant plasmids is greatly affected by culture conditions [33]. Herein, we developed genetically engineered strains to investigate BC biosynthesis in *E. coli* platforms and examined the effect of temperature and inducer concentration on the plasmid stability, the cell growth, and the BC biosynthesis of these strains.

## Materials and methods

### Bacterial strains and plasmids

Bacterial strains and plasmids used in this study are described in Table 1.

**Table 1:** Strains and plasmids used in this study

*Escherichia coli* strains	Feature	Synthesized proteins recombinant proteins	Source
BL21 (DE3)	Native strain	–	Qiagen
GM BL21 (DE3)	Genetically modified strain carrying pBCS and pCMP	BcsA, BcsB, BcsC, BcsD, Cmcax, CcpAx	This study
HMS174 (DE3)	Native strain	–	Qiagen
GM HMS174 (DE3)	Genetically modified strain carrying pBCS and pCMP	BcsA, BcsB, BcsC, BcsD, Cmcax, CcpAx	This study
C41 (DE3)	Native strain	–	Qiagen
GM C41 (DE3)	Genetically modified strain carrying pBCS and pCMP	BcsA, BcsB, BcsC, BcsD, Cmcax, CcpAx	This study

Plasmids	Feature	Inserted genes	Source
pCDFDuet-1	Streptomycin resistant plasmid carrying T7/lac promoter and CloDF13 replicon	–	Novagen
pETDuet-1	Ampicillin resistant plasmid carrying T7/lac promoter and ColE1 replicon	–	Novagen
pCMP	Streptomycin resistant plasmid carrying T7/lac promoter for each inserted gene and CloDF13 replicon	*cmcax, ccp* *A* *x*	This study
pBCS	Ampicillin resistant plasmid carrying T7/lac promoter for each inserted gene and ColE1 replicon	*bcs*A, *bcs*B, *bcs*C, *bcs*D	This study

### Construction of expression vectors

The co-expression system was developed using backbones of two compatible Duet Vectors (Novagen): petDuet-1 and pCDFDuet-1. pETDuet-1 has the ColE1 replicon and *bla* gene for ampicillin/carbenicillin resistance, and pCDFDuet-1 has the CloDF13 replicon and *aadA* gene for streptomycin resistance. Each of these vectors was designed to express multiple genes and each gene was controlled by a separate T7*lac* promoter that provides a tight control of the expression by IPTG (isopropyl β-D-1-thiogalactopyranoside) induction. Each expression unit has own optimal ribosomal binding side located downstream of the promoter. The sequences for *cmcax, ccpAx, bcs*A, *bcs*B, *bcs*C, and *bcs*D of *G. hansenii* ATCC 53582 were obtained from literature. Access numbers for the upstream region (*cmcax* and *ccpAx*) and *bcs*ABCD operon are AB091058 and X54676, respectively [12, 34]. In an effort to enhance the efficiency and accuracy of translation, these target sequences were optimized according to codon usage of *E. coli* by Genewiz, Inc.

**Fig. 1:**
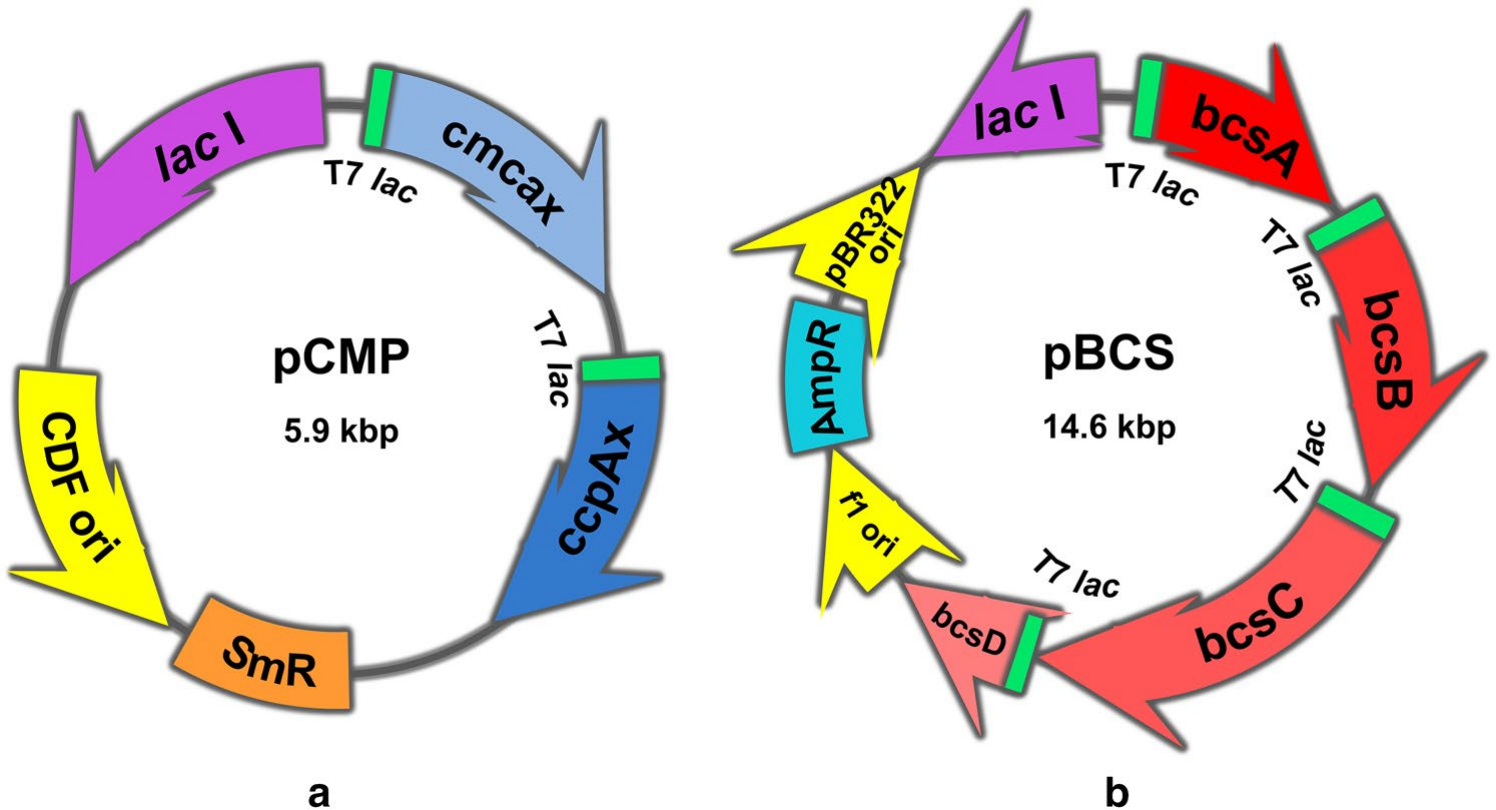
Strategy used for the construction of pCMP (**a**) and pBCS (**b**). pCMP expression unit for the upstream operon (*cmcax* and *cpp*Ax) with T7 promoter and *lac* operator, streptomycin resistance region, CloDF13 replicon (CDF ori), *lac* I region. pBCS expression unit for *bcs* operon with T7 promoter and *lac* operator (*bcs*A, *bcs*B, *bcs*C, and *bcs*C), f1 replicon, ampicillin resistance region, pBR322-derived ColE1 replicon, *lac* I region

Cloning was performed by the restriction digest procedure; *ccpAx* was cloned into pCDFDuet-1 via 5′ using BglII and 3′ XhoI as ligation sites, generating the intermediate construct ccpAx_pCDFDuet-1 for the next step. Following that, *cmcax* was inserted into ccpAx_pCDFDuet-1 via 5′ BamHI and 3′ Hindlll as ligation sites to create the final construct pCMP (Fig. 1a). The sequences of *bcs*A and *bcs*B were co-inserted into pETDuet-1 via 5′ BamHI and 3′ AflII to generate the intermediate construct. After that, *bcs*C and *bcs*D were co-inserted into this intermediate construct via 5′ FseI and 3′ XhoI to generate pBCS (Fig. 1b). *E. coli* strains were then transformed with the constructed plasmids by chemical co-transformation. The selection of transformations was accomplished by plating the cells onto LB agar plates supplemented by 25 mg/ml carbenicillin and 25 mg/ml streptomycin (Calbiochem).

### Verification of the inserted fragments in pCMP and pBCS

To verify whether the target fragments were cloned in the correct orientations, the restriction enzyme digestion tests were performed for both plasmids. BamH I and Hind III cleaved pCMP at two edges of the sequences encoding *cmcax*, and resulted in two linear fragments with the length of 1042 and 4819 bp. Mlu I cleaved the pBCS from two sites that were located on the sequences of bcsC and lacI genes, while Xho I cleaved it at the end of the sequence encoding bcsD on this plasmid. These generated three linear fragments with length of 3071, 4304, and 7219 bp. The fragments are visualized on agarose gel to detect the DNA bands corresponded to their size. Unrestricted plasmids were also visualized to verify the total size, which also showed bands corresponding to the total size of pCMP (5.9 kbp) and pBCS (14.6 kbp) (Online Resource, Fig. ESM1).

### Fermentation of genetically modified (GM) *E. coli* cells

GM *E. coli* pre-cultures were cultured in flasks at 37 °C and 180 rpm, in Lysogeny Broth (LB) medium [10 g/L tryptone (Sigma), 5 g/L yeast extract (Sigma), 10 g/L NaCl (Sigma)]. The culture media were supplemented by both streptomycin (50 mg/ml) and carbenicillin (50 mg/mL) for the maintenance of pCMP and pBCS, respectively. Although pBCS carries a β-lactamase (*bla*) gene, which confers resistance to ampicillin, also confers resistance to carbenicillin. Carbenicillin was used in GM cultures, because it tends to be more stable than ampicillin. 4 g/L glucose (Sigma) was used as a culture supplement not only to prevent basal expression of recombinant proteins prior to the IPTG induction but also as a carbon source for BC biosynthesis. The simultaneous expression of recombinant BC proteins in genetically modified (GM) *E. coli* strains was initiated by inducing the strong T7*lac* promoter with IPTG. Following the IPTG induction, the culture temperature was shifted to 30 °C or 22 °C, if necessary. The effect of IPTG induction on plasmid stability and BC production in each GM cell line was examined at different culture temperatures (22 °C, 30 °C, and 37 °C). The experimental conditions used in this study are summarized in Online Resource, Table ESM1. Optical density (OD) of the cells was measured at 600 nm using spectrophotometer to monitor cell growth.

### Determination of Dry Cell Weight (DCW)

GM cells were cultured overnight in LB medium at 180 rpm and 37 °C. The cells were then centrifuged at 5000 *x g* for 10 min and washed with distilled water for three times, and 50 ml of cells were re-suspended in 100 ml of distilled water and dried at 80 °C. Cell dry weight (mg/ml) for each sample was determined. The remaining 50 mL of cells were used to prepare diluted cell culture samples; OD_600_ nm values of the samples were obtained a spectrophotometer. A calibration curve was prepared by plotting optical density vs. DCW (g/L).

### Plasmid stability test

Plasmid-harboring GM cells were distinguished from the total viable cells using the plasmid-borne antibiotic resistance. The diluted samples were plated on selective (supplemented with 50 mg/mL streptomycin and 50 mg/mL carbenicillin) and non-selective (without antibiotics) LB plates. The colony forming units (cfu) were counted after cultivation at 37 °C overnight [30]. The percentage of plasmid-harboring colonies among total viable colonies on the plates represents the plasmid stability.

### q-PCR assays

The culture samples were collected at specific time intervals by centrifugation at 10,000×*g* for 10 min at 4 °C. The samples were treated with RNAprotect Bacteria Reagent (Qiagen) to stabilize RNA before extraction of RNA. RNeasy Mini Kit (Qiagen) was used to purify the total RNA. The QuantiTect Reverse Transcription (Qiagen) protocol was followed for the synthesis of cDNA. q-PCR analysis was conducted by following QuantiTect SYBR Green Protocol [35]. Total reaction volume was 20 µL, which contained 12.5 mL SYBR Green PCR Master Mix, 0.5 mL of forward primer (0.3 µM) and 0.5 mL of reverse primer (0.3 µM) (Invitrogen, UK), 1 µl of cDNA (100 ng), and 5.5 mL of sterile water. q-PCR analysis was conducted in duplicate measurements for each time point. Gradient PCR was performed to select the annealing temperature for the primer pairs. A melting curve was generated for each reaction to ensure the specificity of each PCR product. *ser*C was used as housekeeping gene [36]. Negative controls (no template controls) were used to serve as a general control for nucleic acid contamination and primer dimer formation. The un-induced cells right before the IPTG induction (*T* = 0) as positive controls were used as positive control to monitor the response of the GM cells to IPTG. Then, the data were normalised to the expression levels in positive controls.

The denaturation temperature was set at 95 °C for 3 min, followed by 95 °C for 20 s. In the next step, different annealing temperatures were employed in each column of the thermocycler. The annealing temperatures ranged from 58 to 60 °C for 30 s, and 72 °C for 30 s. The amplification last for 50 cycles. The oligonucleotide sequences used in this study can be found in Online Resources, Table ESM2.

### Extraction of proteins and SDS PAGE analysis

Periplasmic proteins were extracted by the osmotic shock procedure [37]. In cases where induction with IPTG was needed, GM *E. coli* cells were grown until the optical density at 600 nm of the cell culture reached 1.0 and 0.2 mM IPTG was added to culture medium. Grown cells were harvested by centrifugation for 10 min at 5000 *x g*, 4 °C and re-suspended in 20% sucrose solution (w/v) in 30 mM Tris–HCl (pH 8.0) with 1 mM ethylenediaminetetraacetic acid (EDTA). Following a second centrifugation at 10,000*×g* for 20 min, the pellet was rapidly re-suspended in ice-cold 5 mM MgCl_2_ and gently incubated for 20 min. Cells were removed from the periplasmic protein extract by centrifugation at 10,000*×g*, 4 °C for 20 min. Supernatant contained the periplasmic proteins.

The insoluble fractions were extracted by treatments with BugBuster Reagent (Sigma). rLysozym solution was added to a final concentration of 1 KU/ml. The suspension was centrifuged at 5000*g* for 15 min at 4 °C to collect the insoluble fraction. After re-suspension of the insoluble fractions in half of the original culture volume of 1:10 diluted BugBuster, the sample was again mixed by vortex and centrifuged as in previous step. This step was repeated a few times. The sample was centrifuged at 16,000*g* for 15 min at 4 °C and the supernatant removed. Protein concentrations of the extracted proteins were determined using the method described by Bradford (1976) prior to the SDS PAGE analysis [38].

Protein samples were mixed with 2× Sample Buffer containing SDS at a 1:1 ratio and boiled for 5 min to denature the proteins. The marker and the samples were loaded into the wells of 12% SDS polyacrylamide gels. Separation of proteins based on size was achieved by allowing them to run at 120 V for approximately 90 min.

### Recovery of bacterial cellulose

Following 24 h culturing, the cultures first filtered to recover the BC particles. The samples were treated with 1% NaOH solution (w/v) at 70 °C for 20 min to remove bacteria and other impurities. The samples were then rinsed with deionized water several times until neutral pH and then dried in microcentrifuge tubes at 50◦C to constant weight to obtain dry weight.

### Scanning electron microscopy (SEM) analysis

The morphology and the microstructural features of samples produced by GM *E. coli* strains were investigated using the field-emission scanning electron microscope (FESEM) LEO 1525 (Zeiss, Germany) operating at 5 kV. Prior to analysis, samples were dried until constant weight and placed onto double-sided carbon tape mounted onto an aluminum stub. They were gold coated for 2 min at 20 mA using an Emitech K575X Peltier (Ashford, UK) cooled sputter coater.

### Fourier transform infrared (FTIR) analysis

FTIR spectroscopy was used to characterize cellulose samples produced by bacteria. ATR-IR spectra were recorded using a Spectrum One FTIR-spectrometer (Perkin Elmer, Massachusetts, USA). The spectra were collected at a resolution of 2 cm^−1^ in the range of 600 and 4000 cm^−1^.

### Statistical analysis

Each fermentation was repeated three independent times and each analysis was replicated at three times. Statistical analysis was performed using one-way analysis of variance (ANOVA) and statistical significance was assigned to *P* < 0.05.

### X-ray diffraction (XRD) analysis

Crystallographic analysis of the samples was conducted using a Philips X’Pert Pro diffractometer (PANalytical, The Netherlands) equipped with a Nickel-filtered Cu-Kα radiation (*λ* = 1.5406 Å). The operating voltage and current were 40 kV and 40 mA, respectively. The diffraction spectra were collected over a range of 2*θ* values from 5° to 40° in increments of 0.04°. The relative crystallinity index was calculated by Segal’s method [39]:

$$Cr=100 \times \frac{{\left( {{I_{002}} - {I_{{\text{am}}}}} \right)}}{{{I_{002}}}},$$

where *I*_002_ is the peak intensity corresponding to the (110) plane and I_am_ is the peak intensity of amorphous fraction at 2*θ* = 18°.

## Results

### Optimization of IPTG concentration and temperature for BC biosynthesis

In plasmid-based expression systems, plasmid replication and maintenance can be easily affected by the inducer concentration and the fermentation temperature. Consequently, these factors can severely affect the process outcome. Appearance of plasmid-free cells is not totally prevented even when antibiotic selection is used, as the concentration of antibiotics often decreases during long-term cultivation as a result of dilution and/or enzymatic degradation by the growing cells [30, 40–43].

All IPTG-induced groups of GM BL21 (DE3) exhibited a dramatic decrease in plasmid stability compared to un-induced GM cells within 1 h after the induction at both 37 °C and at 30 °C (*P* < 0.05) (Online Resource, Fig. ESM2, a&b). The use of elevated growth temperatures (> 30 °C) has been previously described as one of the reasons for plasmid instability [44]. However, the final plasmid stability of the induced cells was calculated to be only 5% at 22 °C and production of extracellular BC was not detected in GM BL21 (DE3) under any conditions (Online Resource, Fig. ESM2, c).

**Fig. 2:**
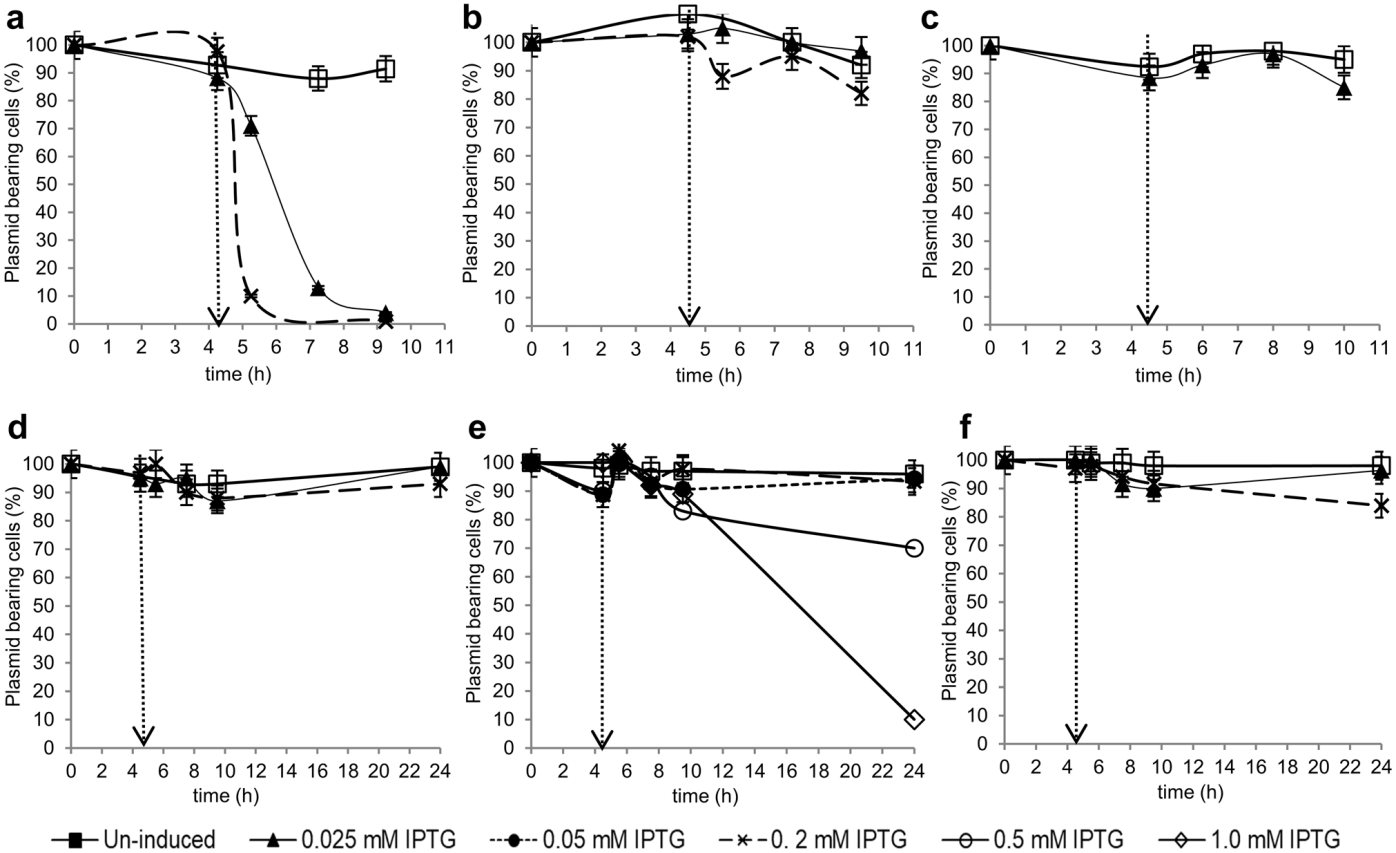
Effect of IPTG induction on plasmid stability in HMS174 (DE3) at 37 °C (**a**), 30 °C (**b**), and 22 °C (**c**), and in GM C41 (DE3) at 37 °C (**c**), 30 °C (**d**), and 22 °C (**f**). The dashed arrows indicate IPTG addition

When induced GM HMS174 (DE3) cells were cultured at 30 °C, the plasmid stability was significantly improved compared that of 37 °C (Fig. 2a, b). The final percentage of plasmid-bearing cells induced by 0.025 mM IPTG and 0.2 mM IPTG was determined to be 97 and 82%, respectively. Nevertheless, BC production was not detected at 30 °C. The plasmid stability in this GM strain remained above 85% at 22 °C (0.025 mM IPTG) and BC formation was detected with a concentration of 23.0 ± 0.02 mg/L (Fig. 2c). This suggested that the effect of the metabolic stress caused by recombinant protein expression was eliminated by lowering both the culture temperature and the IPTG concentration. Therefore, BC biosynthesis in GM C41 (DE3) was investigated at 22 °C initially (Fig. 2f). The GM C41 (DE3) cells induced by 0.2 mM IPTG produced 8.5 ± 0.03 mg/L cellulose (83% plasmid-bearing), while those induced by 0.025 mM IPTG produced 13.5 ± 0.02 mg/L (96% plasmid-bearing). As C41 (DE3) has been proved previously that it was a superior strain in overexpression of membrane proteins and plasmid stability compared to other *E. coli* strains, higher IPTG concentrations were also examined [28–30]. At 30 °C, BC production in the cultures induced by 0.05 mM IPTG and by 0.2 mM IPTG (both > 90% plasmid-bearing) was 21.0 ± 0.02 mg/L and 31.1 ± 0.03 mg/L, respectively. Increasing the concentration of IPTG to 1.0 mM did not stimulate BC production, because the plasmid stability dropped dramatically (Fig. 2e). At 37 °C, the final plasmid stability was calculated to be higher than 93% in both of the induced cultures (Fig. 2d). However, BC production was not observed in any of the GM C41 (DE3) cells cultured above 30 °C or induced by above 0.2 mM IPTG, proving that high IPTG concentrations/temperatures contribute to the stress level of the host.

The growth kinetics of the induced GM lines was slightly slower compared to the un-induced GM lines at 37 °C. However, lowering the culture temperature (to 30 or 22 °C) closed the gap between the growth of the un-induced and the induced groups (data not shown). Dry cell weights (DCW) and product concentrations are presented in Table 2 under the conditions that BC production was detected. GM C41 (DE3) was identified as the best performer, as it exhibited the highest productivity with 34.3 ± 0.22 mg cellulose/g cells.

**Table 2:** Dry cell weight (DCW), product concentration (mg/L), and productivity (mg cellulose/g cells) under the conditions that cellulose biosynthesis was detected

GM HMS174 (DE3)	22 °C		0.025 mM IPTG	
		DCW (g/L)	0.94 ± 0.05	
		Product concentration (mg/L) Productivity (mg cellulose/g cells)	23.0 ± 0.02 24.5 ± 0.86	
GM C41 (DE3)	30 °C		0.05 mM IPTG	0.2 mM IPTG
		DCW(g/L)	0.91 ± 0.06	0.78 ± 0.04
		Product concentration (mg/L) Productivity (mg cellulose/g cells)	31.1 ± 0.03 34.3 ± 0.22	21.0 ± 0.02 27.4 ± 0.14
	22 °C		0.025 mM IPTG	0.2 mM IPTG
		DCW(g/L)	0.90 ± 0.05	0.85 ± 0.04
		Product concentration (mg/L) Productivity (mg cellulose/g cells)	8.5 ± 0.03 9.5 ± 0.05	13.5 ± 0.02 15.9 ± 0.07

### Effect of IPTG concentration on the expression of *cmcax, ccpAx*, and *bcs*ABCD

Confirmation of the expression of the recombinant genes (*cmcax, ccpAx, bcs*A, *bcs*B, *bcs*C, and *bcs*D) in GM cells was performed by q-PCR analysis. For GM BL21 (DE3), all target genes highly expressed even 15 min after IPTG induction (Online Resource, Fig. ESM3, a&b). The gene expression levels of GM BL21 (DE3) induced by 0.2 mM IPTG were much higher than those induced by 0.025 mM IPTG (Online Resource, Fig. ESM3, c). For an effective expression of membrane proteins, transcription of expression plasmids should be induced slowly with low concentrations of IPTG to prevent accumulating of inactive target proteins [45–48]. Elevated levels of gene expression in GM BL21 (DE3) suggest that BC may not be detected as a result of aggregation of the encoded proteins as inclusion bodies. The expression of recombinant genes in GM BL21 (DE3) was detected even after the dramatic decrease in plasmid stability (18 h). This could be because the GM cells was retaining only one of the plasmids, which would not allow the expression of the complete target sequence in a single cell for the successful biosynthesis of BC.

Expression of the recombinant genes in GM HMS174 (DE3) was investigated under culture conditions in which BC was detected (0.025 mM IPTG, 22 °C). In contrast to the q-PCR data obtained for BL21 (DE3), the gene expressions in GM HMS174 (DE3) were observed 3 h after induction at lower but significant levels (between 1.41 and 2.71 fold), which is more desirable to allow the expression of target proteins in an active form (Online Resource, Fig. ESM4, a).

Under the conditions that the highest BC production detected (30 °C, 0.05 mM IPTG) in GM C41 (DE3), the gene expressions 3 h after IPTG induction were as low as (0.25–2.12 fold) those detected in GM HMS174 (DE3) (Online Resource, Fig. ESM4, b). After 18 h of induction, *bcs* genes expressed between 1.61- and 2.19-fold, while upstream genes *cmcax* and *ccpAx* expressed 5.02- and 3.32-fold, respectively. All target genes expressed at higher levels in the presence of 1.0 mM IPTG compared to those expressed in the presence of 0.05 mM IPTG as expected (Online Resource, Fig. ESM4, c).

### SDS–PAGE analysis of *bcs* proteins

**Fig. 3:**
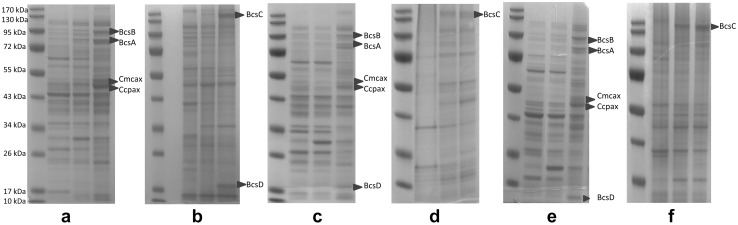
Periplasmic (**a**) and insoluble (**b**) fractions of native BL21 and GM BL21 (DE3) induced by 0.2 mM IPTG. For a&b, Line 1 shows the molecular weight marker, Lines 2, 3, 4 show the extracts of native cells, un-induced GM cells, and induced GM cells (18 h), respectively. Periplasmic (**c**) and insoluble (**d**) fractions of native HMS174 (DE3) and GM HMS174 (DE3) induced by 0.2 mM IPTG. For c&d, Line 1 shows the molecular weight marker, Line 2 shows the extracts of native cells. Lane 3 and Lane 4 show the extracts of induced GM cells collected 3 h and 18 h after the induction. Periplasmic (**e**) and insoluble (**f**) fractions of native C41 (DE3) and GM C41 (DE3) induced by 0.2 mM IPTG. For e&f, Line 1 shows the molecular weight marker, Line 2 shows the extracts of native cells. Lane 3 and Lane 4 show the extracts of induced GM cells collected 3 h and 18 h after the induction

BC is synthetized on the cytoplasmic side of the inner membrane and is transported through the periplasm before being secreted into the extracellular space [49, 50]. Therefore, *bcs* proteins are located in periplasm or in cell membrane [19, 49–53]. Membrane proteins are difficult to solubilize and often found in insoluble protein fraction. Therefore, both periplasmic and insoluble fractions of GM cells were analyzed by SDS PAGE. Two protein bands corresponding to the sizes of BcsC and BcsD were detected in the insoluble fraction of GM BL21 (DE3), while the other proteins were detected in the periplasmic fraction (Fig. 3a, b). Membrane proteins are often difficult to solubilize and most insoluble protein fractions contain membrane proteins along with inclusion bodies [54, 55] Therefore, it is difficult to predict whether BcsC (an outer membrane protein) detected in insoluble fraction was in a form of aggregation or properly folded. Detection of BcsD in the insoluble fraction could be the reason for GM BL21 (DE3) not being able to produce BC, as BcsD is a periplasmic protein that is responsible for the crystallization of BC.

CcpAx and Cmcax were detected slightly above their size region (4 kDa) due to the additional sequences (105 bp including His-tag for *cmcax* and 96 bp for *cppAx*) on the cloning sites of pCMP in upstream of these proteins after translation starts. A protein band corresponding to the size of BcsC, an outer membrane protein, was detected in the insoluble fraction of GM HMS174 (DE3) and GM C41(DE3), whereas the other proteins were detected in periplasmic fraction of both (Fig. 3c–f). Clear bands corresponding to the target proteins were detected when GM cells were induced with 0.2 mM IPTG. However, SDS–PAGE analysis did not reveal a significant difference in between the induced fractions and un-induced fractions in the conditions where lower concentrations of IPTG (0.025, 0.05 mM) resulted into BC synthesis (data not shown). The concentration of target proteins could be below the detection limit of SDS–PAGE analysis due to the slow induction, but still well enough to cause the activity.

### Direct detection of BC in GM *E. coli* HMS174 (DE3) and GM C41 (DE3) cell cultures

**Fig. 4:**
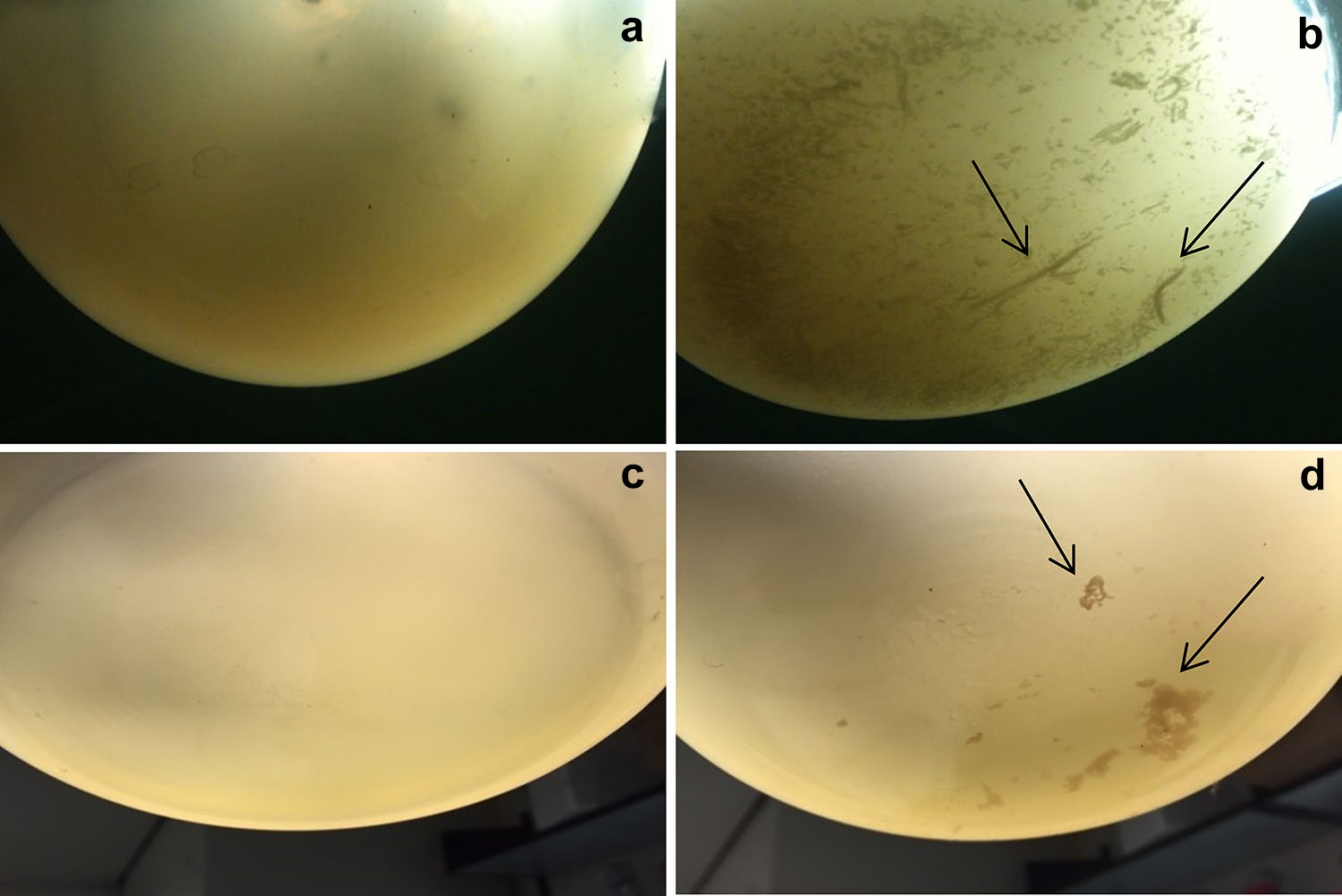
Liquid culture images of native HMS174 (DE3) (**a**), GM HMS174 (DE3) (**b**), native C41 (DE3) (**c**), and GM C41 (DE3) (**d**). GM strains were cultured at 22°C and induced by 0.025 mM IPTG

Since BC is an extracellular material secreted into the culture medium, it was directly detected 18 h after the induction in cultures of HMS174 (DE3). In GM C41 (DE3) cultures, BC production was observed even 3 h after the induction (Fig. 4b, d). BC production was not found in the cultures of native HMS174 (DE3) and native C41 (DE3) used as negative controls (Fig. 4a, c). The shaking speed is one of the most important parameters that can significantly influence the morphology of BC in cell cultures. Typically, BC produced by *G. hansenii* is in the form of a dense and flat gel-like layer that covers the surface of the culture medium under static condition, which is more preferred to avoid the shear stress generating the mutation of *G. hansenii* into non-cellulose-producing state in high agitation [56–58]. Under agitated culture conditions, many particles with various sizes (10 µm to 1 mm) and various shapes (spherical, ellipsoidal, fibrous, or stellate) were generated in BC cultures to form a well-dispersed suspension previously [57, 59, 60]. Here, BC was detected in the form of dispersed fibre-like structures inside the culture media of both GM HMS174 (DE3) and C41 (DE3). A less dense BC was produced by GM strains unlike the structure of original BC produced under agitated conditions. This was possibly due to the fact that BC was exposed to the vigorous agitation in GM cultures not only to provide enough oxygen to the cells but also to prevent the cells from precipitating, as *E. coli* tends to settle to the bottom of cell cultures in the absence of agitation unlike *G. hansenii*.

### Morphology characterization of BC

**Fig. 5:**
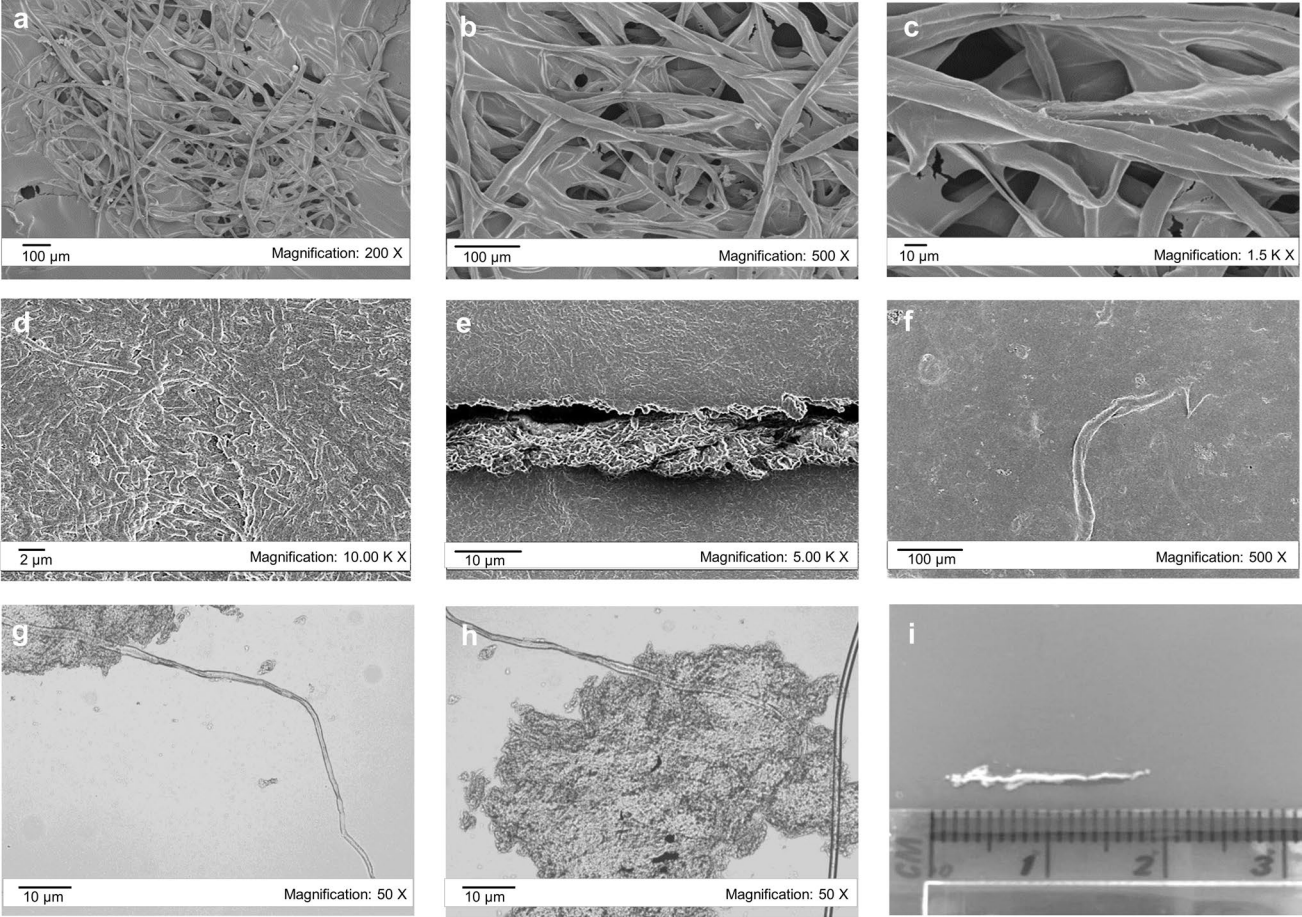
Scanning electron micrographs showing the microstructure of BC produced by GM HMS174 (DE3) (**a**–**c**) and GM C41 (DE3) (**d**–**f**) 18 h after the induction at different resolutions. Light microscope images of BC fibres and cell aggradations 18 h after the induction produced by GM C41 (DE3) induced with 0.05 mM IPTG at different resolutions (**g, h**). Length of BC particles that were produced by GM C41 (DE3) under the same conditions (**i**)

The morphology of BC produced by GM cells was characterized by SEM and light microscopy. SEM images showed that BC produced by GM HMS174 (DE3) has smooth fibres that formed a dense and random network, which is similar to the general morphology of BC produced by *G. hansenii* (Fig. 5a–c). However, the fibres exhibited an extraordinary structure in terms of size. The lengths of the fibres were approximately between 1000 and 3000 μm with diameters ranging from 10 to 20 μm. The diameter of BC produced by *G. hansenii* is in nanoscale, typically ranging from 10 to 100 nm, while its length can be between 100—several 1000 nm [3, 61, 62]. The fibres produced by GM HMS174 (DE3) were extremely longer than those in BC typically produced by *G. hansenii*.

**Fig. 6:**
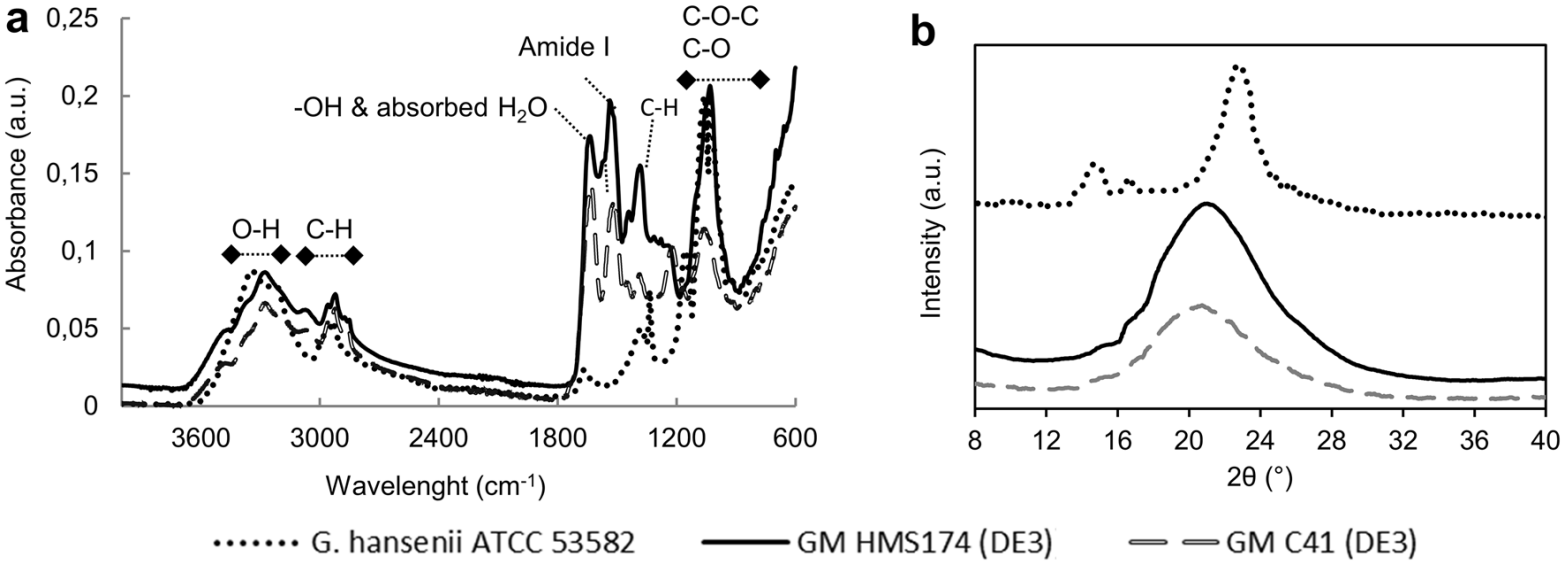
ATR-FTIR spectra (**a**) and XRD (**b**) of BC biosynthesized by *G. hansenii* ATCC 53582, GM HMS174 (DE3), and GM C41 (DE3)

BC produced by GM C41 (DE3) shows a more random network and an irregular pattern compared to that of GM HMS174 (DE3) (Fig. 6d–f). It exhibits a biofilm-like structure, which is rigid, but contains fragile connections between cells [63]. Fibres with a length of approximately 500 μm were detected on SEM images. Microscope images of BC produced by GM C41 (DE3) revealed more clearly that these fibres are embedded into a biofilm-like structure/cell–cell interactions and exhibit a very smooth structure with a length of up to 2 cm (Fig. 5g–i).

### FTIR analysis of BC

The spectra of BC produced by GM strains are characterized by very strong absorption bands located between 900 and 1243 cm^−1^ attributed to the C–O and C–O–C stretching vibration of glucose (Fig. 6a) [64–67]. The band assigned to the stretching of –OH groups present in cellulose is identified between 3500 and 3100 cm^−1^ [68]. The spectral band located between 2800 and 2900 cm^−1^ is assigned to C-H stretching vibrations of BC (including -CH2 and -CH3), whereas the spectral bands located between 1420 and 1278 cm^−1^ are corresponding to the in-plane bending of C-H groups [66, 69]. Cellulose synthesis is correlated with the biofilm formation in *E. coli* [70–72], which is an extracellular polymeric substance composed of extracellular DNA, proteins, and polysaccharides. Another noticeable region of the spectra appeared around 1517 and 1537 cm^−1^, which corresponds to amide I groups in this substance [73]. Furthermore, the band at 1640 cm^−1^ is assigned to –OH bending.

### XRD analysis of BC

The XRD spectra of BC produced by GM strains are presented in Fig. 6b. BC biosynthesized by *G. hansenii* ATCC 53582 exhibits the typical spectrum of cellulose I planes, which correspond to [110], [110], and [200] positioned at 14.5°, 16.6°, and 22.8°, respectively [74–76]. The spectrum of BC produced by GM strains showed one broad band at around 21° along with two weak peaks at 14.4° and 16.6°, which exhibited clearly more amorphous structure. The degree of crystallinity of BC biosynthesized by *G. hansenii* ATCC 53582 is estimated to be as high as 85%, while it was estimated to be 47 and 46% for GM HMS174 (DE3) and GM C41 (DE3), respectively. The ratio of the crystalline and amorphous regions depends on various conditions such as cellulose source, microorganism, medium composition, and fermentation conditions [76]. Accordingly, the absence of sharp peaks in XRD spectrum of BC produced by GM may be due to the differences in export mechanisms of bacterial species or to the fermentation conditions.

## Discussion

New cellulose-producing strains have recently been explored by different approaches. It has been shown that the facultative anaerobe *Enterobacter* sp. FY-07 is able to produce cellulose under aerobic and anaerobic conditions. The gene constitution and arrangement of *Enterobacter* sp. FY-07 BC synthesis gene cluster *bcs*III, comprising *bcs*A, *bcs*B, *bcs*C and *bcs*D, are the same as *G. hansenii* ATCC 53582. This suggests that the same BC synthesis mechanism of *G. hansenii* ATCC 53582 exists in *Enterobacter* sp. FY-07 [77]. Authors obtained cellulose production also in *E. coli* DH5α harboring bcsIII and its upstream; however, the study was focused on BC production in *Enterobacter* sp. FY-07 and level of BC production in *E. coli* DH5α was not examined. When the gene (AKI40_0895) upstream of *bcs*III in *Enterobacter* sp. FY-07 was knocked out, the BC biosynthesis ability of mutant *Enterobacter* sp. FY-07 (Δ hyp) decreased sharply. Similarly, in *G. hansenii* ATCC 53582, the gene in the location corresponding to AKI40_0895 encodes a cellulose complementing factor (CcpAx), which was also essential for BC production. In another study, *bcs* genes were constructed using Gibson assembly and transformed into *Komagataeibacter xylinus* for overexpression [78]. Although there were no significant differences between the growth of wild-type and engineered strains, the engineered *K. xylinus* strains demonstrated faster BC production, generating 2–4-fold higher production compared to wild-type strain. *Komagataeibacter rhaeticus*, which is also a cellulose-producing strain, has been used to develop a modular genetic toolkit that enables rational reprogramming of the cell for controlled BC production recently [79]. In another study, the reconstitution of cellulose synthase in *E. coli* was investigated [80]. However, this study did not achieve complete BC biosynthesis, since the important proteins for crystallization and localization were not included in the developed system: BcsC, BcsD, and CcpAx. In addition, no contracts were codon-optimized to eliminate the variations in codon usage between different bacteria in any of these studies, which can have a significant influence on translation efficiency. Here, we included not only *bcs*ABCD operon but also its upstream operon in separate vector constructs to test the effect of the operons separately. BC production was not detected when only *bcs*ABCD operon was expressed in the initial trials (data not shown).

The recombinant co-expression of complex genetic circuits comes with the cost of low evolutionary stability, especially when most of these proteins are membrane-associated [28, 30, 81, 82]. A number of biological restraints, primarily replication of plasmids and culture conditions, are responsible for limiting the final biomass and the product concentration in such systems. To overcome plasmid instability, genome-based expression systems that integrate the target DNA fragments into the chromosome have been established for *E. coli*; however, plasmid-based systems are still preferred, because the cloning procedures are simpler and faster for such large DNA sequences [26, 83, 84]. In plasmid-based systems, it is necessary to carefully optimize process conditions for continuous and stable production of a recombinant product.

The metabolic stress caused by the maintenance of recombinant plasmids ultimately resulted in plasmid instability in BL21 (DE3) expression systems previously [40–42]. This phenomenon was observed in also GM BL21 (DE3). Decreasing culture temperature and slow induction of the target genes (with lower IPTG concentrations) have been defined as favorable for recombinant expression systems to prevent inclusion body formation [45–47, 85–87]. However, these were not able to stop the dramatic plasmid loss in GM BL21 (DE3), since the overexpression of multiple membrane proteins in BL21 (DE3) is usually toxic and often results in high overexpression yields [26]. The high gene expression proved by q-PCR in GM BL21 (DE3) supports this theory.

A comparative study demonstrated that HMS174 (DE3) exhibited a high plasmid stability, while a dramatic plasmid loss clearly occurred in BL21 (DE3) [26]. In addition, overexpression of many membrane proteins in BL21 (DE3) was not successful, whereas this was achieved in C41 (DE3) with high plasmid stability previously [28, 29]. Herein, stable expression systems (plasmid stability above > 85%) were established in both GM C41 (DE3) and GM HMS174 (DE3) when the bioprocess conditions (temperature and IPTG concentration) were carefully optimized. Both of these GM strains were able to biosynthesize BC at lower temperature (≤ 30 °C) and lower IPTG concentrations (≤ 0.2 mM). Gene expression levels revealed that the target genes were slowly expressed 3 h after IPTG induction in these strains. This suggests that the accumulation of inactive proteins may be eliminated by slow induction to allow the production of active proteins [85, 86]. Due to the absence of significant expression levels of the target protein by SDS–PAGE analysis at low IPTG (0.025, 0.05 mM) also supported the possibility of slow expression (data not shown). The BC produced by GM HMS174 (DE3) revealed a notable structure constituted of dense fibres with a length reaching 3000 µm with a width ranging between 10 and 20 µm. The mechanical properties of BC are affected by the length of the fibres and more importantly the aspect ratio (length/width ratio) of the fibres [88, 89]. Herein, we produced fibres with high aspect ratio (> 150), which are, therefore, potentially suitable for high efficiency composite materials for various applications as high aspect ratio is desirable for reinforcement [88, 90]. BC formation was detected even 3 h after IPTG induction in GM C41 (DE3) which allows shortening the fermentation time for BC production. The BC obtained previously in agitated cultures of *G. hansenii* exhibited a lower degree of crystallinity compared to that of static cultures [60]. Here, the BC produced by GM cells showed also a lower degree of crystallization due to the vigorous agitation (180 rpm).

The volumetric productivity of *G. hansenii* ATCC 53582, the donor organism for *bcs* genes herein, was reported approximately to be between 6.0–18.45 mg/L h previously [34, 91–93]. This is calculated to be 1.73 mg/L h for GM *E. coli* C41 (DE3) and 1.36 mg/L h for GM *E. coli* HMS174 (DE3) in our study (Table 3). Although the volumetric productivity appears lower in GM *E. coli* strains, BC productivities should be reported after normalising to carbon source concentrations in culture media as it highly differs in between *E. coli* and *G. hansenii* cultures (4 g/L glucose supplement in LB medium for GM *E. coli* cultures and 20 g/L of glucose in HS medium for *G. hansenii* cultures). The yield of the cellulose from glucose is calculated to be 0.44 mg/g.h in GM *E. coli* C41 (DE3) and 0.34 mg/g.h in GM *E. coli* HMS174 (DE3). Both were obtained in the range of normalised productivity of *G. hansenii* ATCC 53582 cultures (0.30–0.92 mg/g h). A potent cellulose producer, *Gluconacetobacter xylinum* BRC5, produced 13.88 mg/L h (0.69 mg/g h) cellulose in agitated flasks initially [94]. When *G. xylinum* BRC5 was cultured in fed-batch conditions in a jar bioreactor under both pH and dissolved O_2_ control, the volumetric productivity increased significantly and reached to the highest BC productivity obtained so far with 0.3 g/L h (15 mg/g h) [95]. Therefore, volumetric yields in GM strains should be further investigated when using a bioreactor system with an efficient O_2_ supply and pH control, as *E. coli* cells grows highly fast when compared to acetic acid bacteria which could potentially result in higher yields. In addition, the fermentation period for GM *E. coli* strains was only 18 h, whereas it was reported to be around 168 h for *G. hansenii* cultures, which could be an advantage for the reducing the energy consumption for BC production processes.

**Table 3:** Production of BC in static and agitation cultures of *G. hansenii* ATCC 53582 and GM *E. coli* strains

Strain	Cultivation mode	Culture time (h)	Volumetric productivity (mg/L h)	Normalised productivity (mg/g h)	References
*G. hansenii* ATCC 53,582	Static	672	9.27	0.46	[93]
*G. hansenii* ATCC 53,582	Static	168	16.25	0.81	[92]
*G. hansenii* ATCC 53,582	Static	168	18.45	0.92	[91]
*G. hansenii* ATCC 53,582	Agitated	168	16.07	0.80	[91]
*G. hansenii* ATCC 53,582	Static/agitated flasks	168	6.00	0.30	[34]
GM *E. coli* C41 (DE3)	Agitated flasks	18	1.73	0.44	This study
GM *E. coli* HMS174 (DE3)	Agitated flasks	18	1.36	0.34	This study

## Conclusion

We achieved recombinant biosynthesis of bacterial cellulose in *E. coli* platforms by the co-expression of *bcs*ABCD and its upstream operon. The optimization of bioprocess conditions resulted in the functional and stable biosynthesis of BC with remarkable fibre structure, as early as 3 h after the induction. The system developed in this study can be potentially used to contribute to future bioprocess design for bacterial cellulose production by further investigations.

## Electronic supplementary material

Below is the link to the electronic supplementary material.

Supplementary material 1 (DOCX 6227 KB)

## Abbreviations

BC: Bacterial cellulose
*Bcs*: Bacterial cellulose synthesis
Bp: Base pair
DCW: Dry cell weight
FTIR: Fourier transform infrared
GM: Genetically modified
IPTG: Isopropyl β-D-1-thiogalactopyranoside
KU: Kilo unit
Kbp: Kilo base pair
LB Medium: Lysogeny broth medium
q-PCR: Quantitative polymerase chain reaction
RQ: Relative quantification
SEM: Scanning electron microscopy
