# Supplementary material for: Recombinant biosynthesis of bacterial cellulose in genetically modified Escherichia coli
Source: Bioprocess Biosyst Eng. 2017 Nov 24;41(2):265–79. doi: 10.1007/s00449-017-1864-1 (PMC5773641; doi:10.1007/s00449-017-1864-1)
Supplement: Supplementary file 1 — Supplementary material 1 (DOCX 6227 KB) [file 449_2017_1864_MOESM1_ESM.docx]

**Electronic Supplementary Material for**

**Recombinant biosynthesis of bacterial cellulose in genetically modified *Escherichia coli***

*Gizem Buldum^1,4^, Alexander Bismarck^2,3^, Athanasios Mantalaris^1,*^*

^1^ *Biological Systems Engineering Laboratory* (BSEL), Department of Chemical Engineering, Imperial College London, South Kensington Campus, London SW7 2AZ, United Kingdom

^2^ *Polymer and Composite Engineering* (PaCE) Group, Department of Chemical Engineering, Imperial College London, South Kensington Campus, London SW7 2AZ, United Kingdom

^3^ *Polymer and Composite Engineering* (PaCE) Group, Institute of Materials Chemistry & Research, Faculty of Chemistry, University of Vienna, Währinger Str. 42, A-1090 Vienna, Austria.

^4^ Department of Bioengineering, Marmara University, Göztepe Campus, Istanbul, Turkey

*Corresponding author: Athanasios Mantalaris: [a.mantalaris@imperial.ac.uk](mailto:a.mantalaris@imperial.ac.uk)

Tel: +44 (0)20 7594 5601

**List of Tables**

Table ESM1 Experimental conditions used in this study.

Table ESM2 Primers used for q-PCR in this study.

**List of Figures**

**Fig. ESM1** Restriction digest test for pCMP (a) and pBCS (b).

**Fig. ESM2** The effect of IPTG induction on plasmid stability in GM BL21 (DE3) at 37˚C (a), 30 ˚C (b), 22 ˚C (c).

**Fig ESM3** Relative gene expressions of GM BL21 (DE3): (a) 15, 30, 60 min after induction by 0.025 mM IPTG 30˚C; (b) 15, 30, 60 min after induction by 0.2 mM IPTG at 30˚C; (c) 3, 6, 18 h after induction by 0.2 mM IPTG 30˚C.

**Fig ESM4** Relative gene expressions of GM HMS174 (DE3) and GM C41 (DE3): (a) 3 and 18 h after induction of GM HMS174 (DE3) by 0.025 mM IPTG at 22˚C, (b) 3, 6 and 18 h after induction of GM C41 (DE3) by 0.05 mM IPTG at 30˚C, (c) 3, 6 and 18 h after induction of GM C41 (DE3) by 1.0 mM IPTG at 30˚C.

| **Strain** | **Temperature (C)** | **IPTG Concentration (mM)** |
| --- | --- | --- |
| GM BL21 (DE3) | 37 | 0025, 0.05, 0.1, 0.2 |
|  | 30 | 0025, 0.05, 0.1 |
|  | 22 | 0.025 |
| GM HMS174 (DE3) | 37 | 0.025, 0.2 |
|  | 30 | 0.025, 0.2 |
|  | 22 | 0.025 |
| GM C41 (DE3) | 37 | 0.025, 0.2 |
|  | 30 | 0.05, 0.2, 0.5, 1.0 |
|  | 22 | 0.025, 0.2 |

Table ESM1 Experimental conditions used in this study.

Table ESM2 Primers used for q-PCR in this study.

| **Primers** | **Oligonucleotide sequence** | |
| --- | --- | --- |
| \| cmcax (F) \| TGCGACTCCTGCATTAGGAAATTA \| \| --- \| --- \| \| cmcax (R) \| TGGCTGCCATCACACTCATCG \| \|  \|  \| \| cppAx (F) \| TGTACACGGCCGCATAATCG \| \| cppAx (R) \| CACTACCGCTGGCGCTCAT \| \|  \|  \| \| bcsA (F) \| TCCCGCGAAATTAATACGACTCACT \| \| bcsA (R) \| ATCGGATCCTGGCTGTGGTGA \| \|  \|  \| \| bcsB (F) \| TGGCAGTGCCCGGTGTAAT \| \| bcsB(R) \| GTGCGCTTGCTGCTTGTGC \| \|  \|  \| \| bcsC (F) \| ACACGGCCGCATAATCGAAAT \| \| bcsC (R) \| GCGTAGCGCTTGTGTGTCAT \| \|  \|  \| \| bcsD (F) \| TGATTAGCGCCCACTACCTGA \| \| bcsD (R) \| GGAACAGGGTGAAGTCCGGC \| \|  \|  \| \| serC (F) \| GCGCGGTTAAGCCAATGCGT \| \| serC (R) \| GCCGAAGTCTGGCGTCTCGT \| | |  |


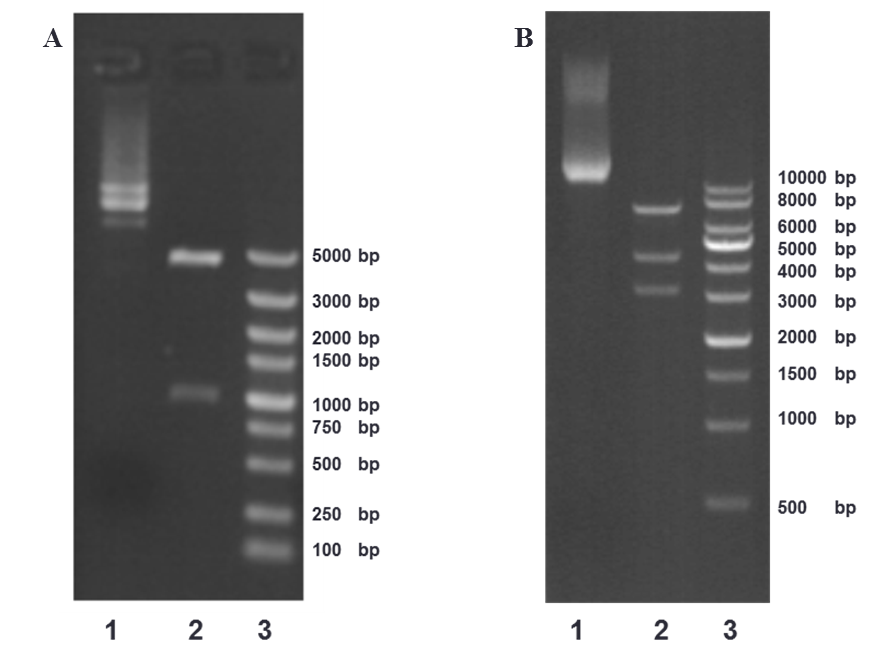


**a b**

**Fig. ESM1** Restriction digest test for pCMP (a) and pBCS (b). For both gel images, Lane1: undigested, Lane 2: digested, Lane 3: DNA ladder.

**
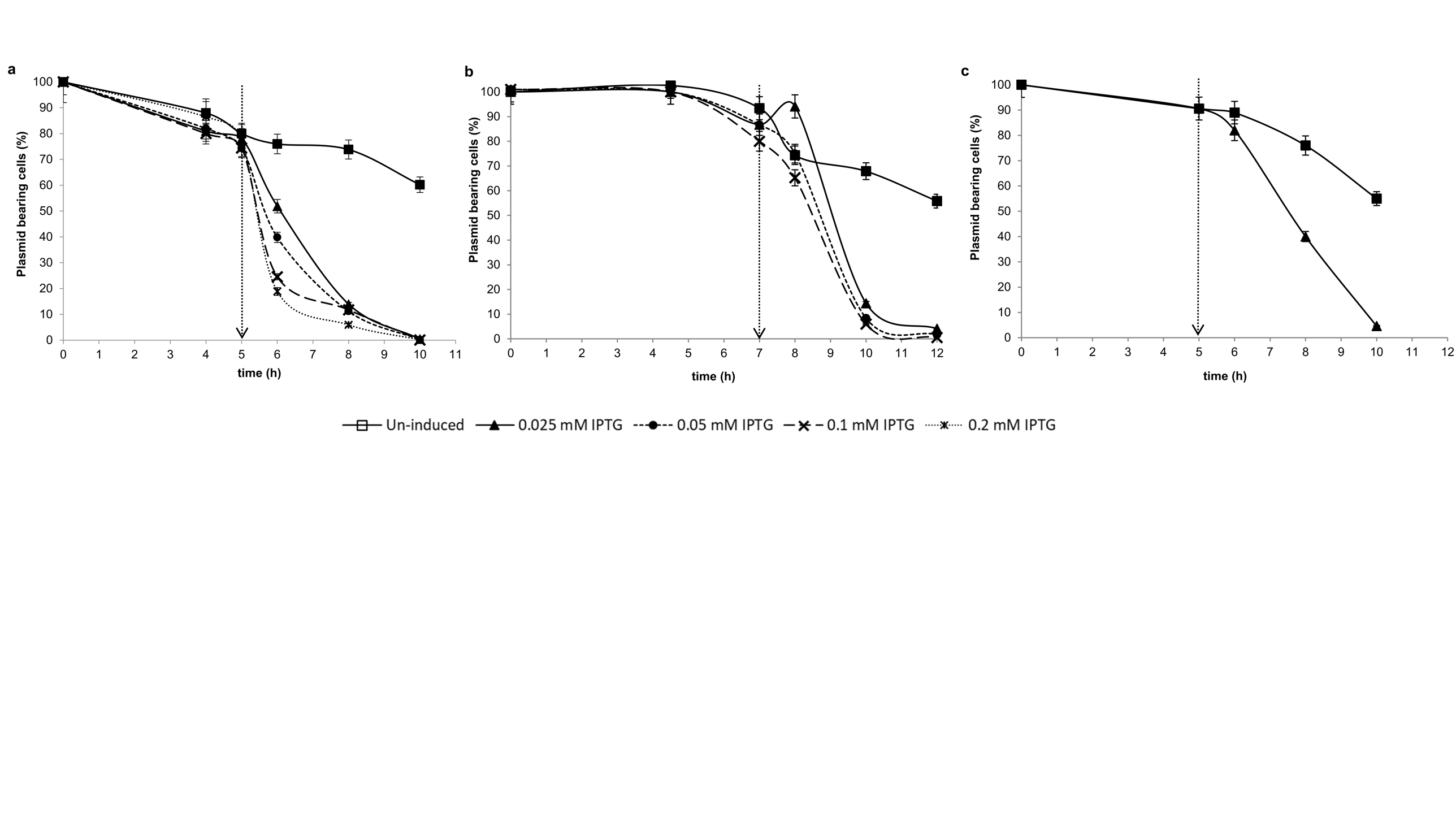
**

**Fig. ESM2** The effect of IPTG induction on plasmid stability in GM BL21 (DE3) at 37˚C (a), 30 ˚C (b), 22 ˚C (c). The dashed arrows indicate IPTG addition.


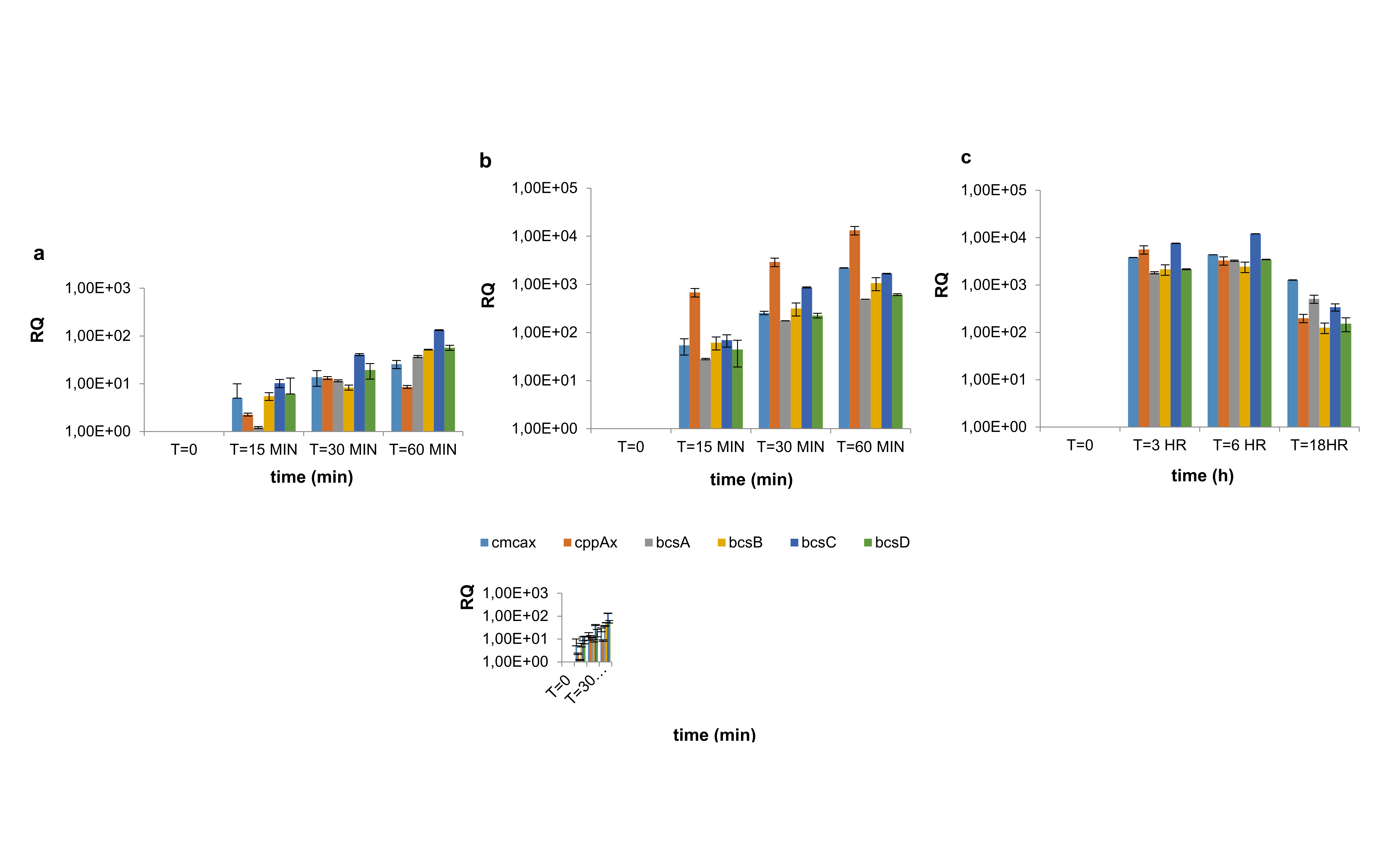


**Fig. ESM3** Relative gene expressions in GM BL21 (DE3): (a) 15, 30, 60 min after induction by 0.025 mM IPTG 30˚C; (b) 15, 30, 60 min after induction by 0.2 mM IPTG at 30˚C; (c) 3, 6, 18 h after induction by 0.2 mM IPTG 30˚C.


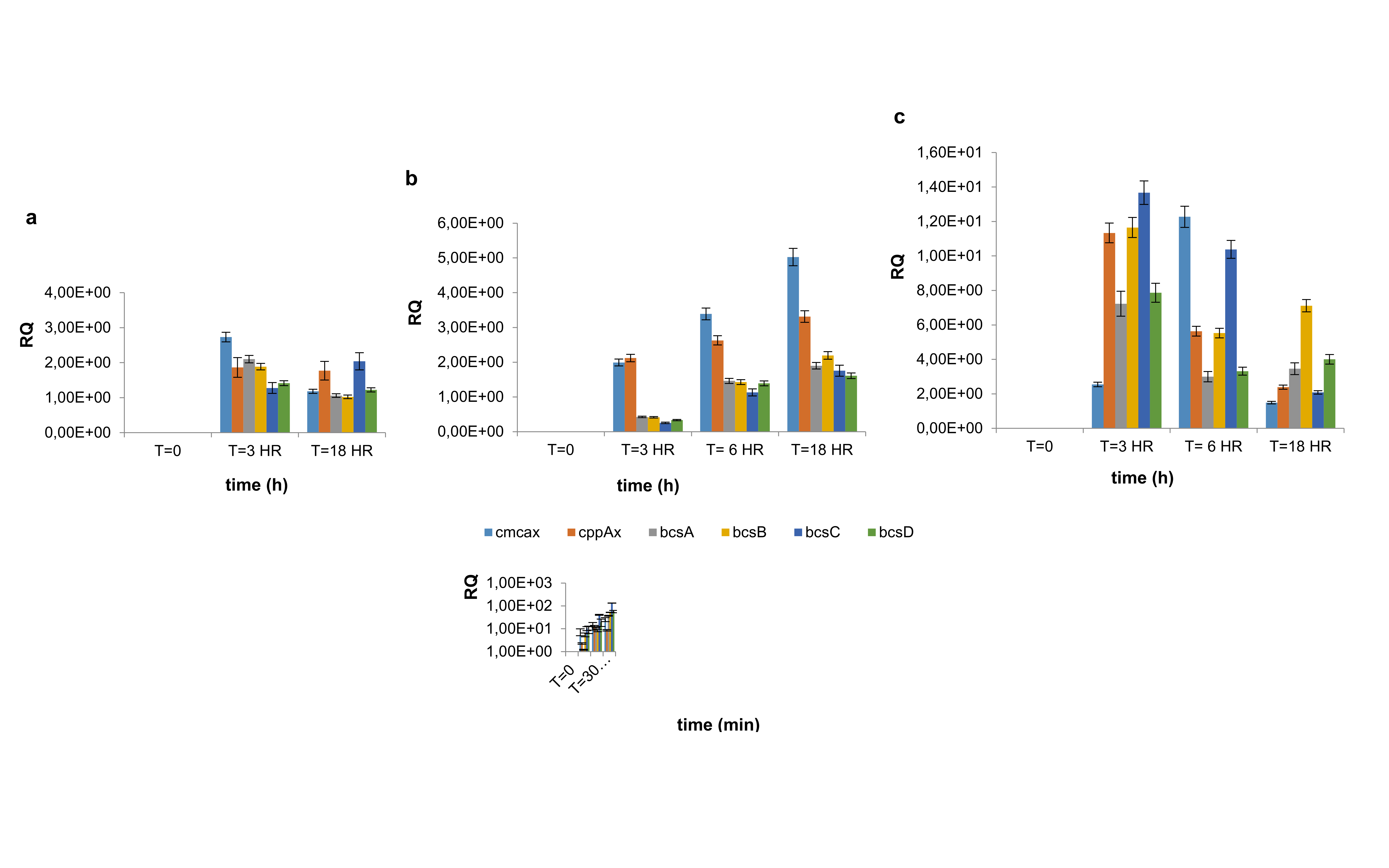


**Fig. ESM4** Relative gene expressions of GM HMS174 (DE3) and GM C41 (DE3) after induction: (a) 3 and 18 h after induction of GM HMS174 (DE3) by 0.025 mM IPTG at 22˚C, (b) 3, 6 and 18 h after induction of GM C41 (DE3) by 0.05 mM IPTG at 30˚C, (c) 3, 6 and 18 h after induction of GM C41 (DE3) by 1.0 mM IPTG at 30˚C.
